# Sensitivity Tests of Pellets Made from Manganese Antimonate Nanoparticles in Carbon Monoxide and Propane Atmospheres

**DOI:** 10.3390/s18072299

**Published:** 2018-07-16

**Authors:** Héctor Guillén-Bonilla, Verónica-M. Rodríguez-Betancourtt, José Trinidad Guillen-Bonilla, Lorenzo Gildo-Ortiz, Alex Guillen-Bonilla, Y. L. Casallas-Moreno, Oscar Blanco-Alonso, Juan Reyes-Gómez

**Affiliations:** 1 Departamento de Ingeniería de Proyectos, Centro Universitario de Ciencias Exactas e Ingenierías (CUCEI), Universidad de Guadalajara, Guadalajara 44410, Mexico; hguillenbonilla@gmail.com; 2 Departamento de Química, Centro Universitario de Ciencias Exactas e Ingenierías (CUCEI), Universidad de Guadalajara, Guadalajara 44410, Mexico; 3 Departamento de Electrónica y Computación, Centro Universitario de Ciencias Exactas e Ingenierías (CUCEI), Universidad de Guadalajara, Guadalajara 44410, Mexico; jtgbonilla@gmail.com; 4 Departamento de Física, Centro Universitario de Ciencias Exactas e Ingenierías (CUCEI), Universidad de Guadalajara, Guadalajara 44410, Mexico; lorenzo.gildo@gmail.com (L.G.-O.); oscar.blanco@cucei.udg.mx (O.B.-A.); 5 Departamento de Ciencias Computacionales e Ingenierías, Centro Universitario de los Valles (CUVALLES), Universidad de Guadalajara, Carretera Guadalajara-Ameca Km 45.5, Ameca 46600, Mexico; alexguillenbonilla@gmail.com; 6 Consejo Nacional de Ciencia y Tecnología (CONACYT), Instituto Politécnico Nacional—UPIITA, Av. IPN 2580 Col. Barrio la Laguna Ticomán, Ciudad de Mexico C.P. 07340, Mexico; yenycasallas@gmail.com; 7 Facultad de Ciencias, Universidad de Colima, Las Víboras 28045, Mexico; reyesgj@ucol.mx

**Keywords:** MnSb_2_O_6_, nanoparticles, pellets, sensitivity, gas sensor

## Abstract

Nanoparticles of manganese antimonate (MnSb_2_O_6_) were prepared using the microwave-assisted colloidal method for its potential application as a gas sensor. For the synthesis of the oxide, manganese nitrate, antimony chloride, ethylenediamine and ethyl alcohol (as a solvent) were used. The precursor material was calcined at 800 °C in air and analyzed by X-ray diffraction. The oxide crystallized into a hexagonal structure with spatial group P321 and cell parameters a = b = 8.8054 Å and c = 4.7229 Å. The microstructure of the material was analyzed by scanning electron microscopy (SEM), finding the growth of microrods with a size of around ~10.27 μm and some other particles with an average size of ~1.3 μm. Photoacoustic spectroscopy (PAS) studies showed that the optical energy band (*Eg*) of the oxide was of ~1.79 eV. Transmission electron microscopy (TEM) analyses indicated that the size of the nanoparticles was of ~29.5 nm on average. The surface area of the powders was estimated at 14.6 m^2^/g by the Brunauer–Emmett–Teller (BET) method. Pellets prepared from the nanoparticles were tested in carbon monoxide (CO) and propane (C_3_H_8_) atmospheres at different concentrations (0–500 ppm) and operating temperatures (100, 200 and 300 °C). The pellets were very sensitive to changes in gas concentration and temperature: the response of the material rose as the concentration and temperature increased. The results showed that the MnSb_2_O_6_ nanoparticles can be a good candidate to be used as a novel gas sensor.

## 1. Introduction

The oxides of transition metals—known as antimonates—have recently attracted the attention of several research groups due to their interesting physical and chemical properties [1]. These materials have been used in different technological areas (like in photocatalytic degradation processes, thermoelectric conversion materials, as negative electrodes in rechargeable lithium-ion batteries, etc.) because of the type of microstructure obtained during their synthesis process [2,3,4]. In recent years, antimonates like CoSb_2_O_6_, NiSb_2_O_6_, MgSb_2_O_6_ and ZnSb_2_O_6_ have been used as potential gas sensors in CO_2_, CO, C_3_H_8_, LPG, H_2_S and NO_2_ atmospheres [5,6,7,8,9,10,11,12]. It has been found that such materials possess a high response, which is attributed to the morphology, porosity and nanometric particle size obtained during their preparation [5,6,8,9].

The antimonates, mainly the trirutile-type ones, conform to the stoichiometric formula ASb_2_O_6_, where A can be substituted by divalent ions such as Co, Ni, Mg, Zn, Cu, Mn, Ca, or Sr [1,13,14]. The substitution of these ions during the synthesis yields trirutile-type crystalline structures with spatial groups P4_2_/mnm (MgSb_2_O_6_, NiSb_2_O_6_, CoSb_2_O_6_) [9,15,16] and P2_1_/n or P2_1_/c (CuSb_2_O_6_) [17]. Some authors state that substituting the Mn ion (in order to obtain the manganese antimonate, MnSb_2_O_6_), the compound may show a hexagonal or columbite-type crystal structure with spatial group P321 [18], displaying also a strong similarity to the trirutile-type structures of the FeSb_2_O_6_, the CoSb_2_O_6_ and the NiSb_2_O_6_ [19]. Other authors have reported that the manganese antimonate can show a trigonal chiral structure [20]. Typically, these oxides are synthesized by the solid-state reaction route [4]. However, the colloidal method has been employed in recent works because it is a way to have a better control of the physical and chemical parameters in order to obtain particle sizes of nanometric scale [5,7,9,10].

In this work, nanoparticles of manganese antimonate were synthesized following a simple and economical process to obtain particles with sizes less than 100 nm. Pellets were made from powders of the MnSb_2_O_6_ and exposed to atmospheres of propane (C_3_H_8_) and carbon monoxide (CO) at different concentrations and temperatures of operation, showing a high sensitivity.

## 2. Materials and Methods

### 2.1. Synthesis of MnSb_2_O_6_ Nanoparticles

**Figure 1:**
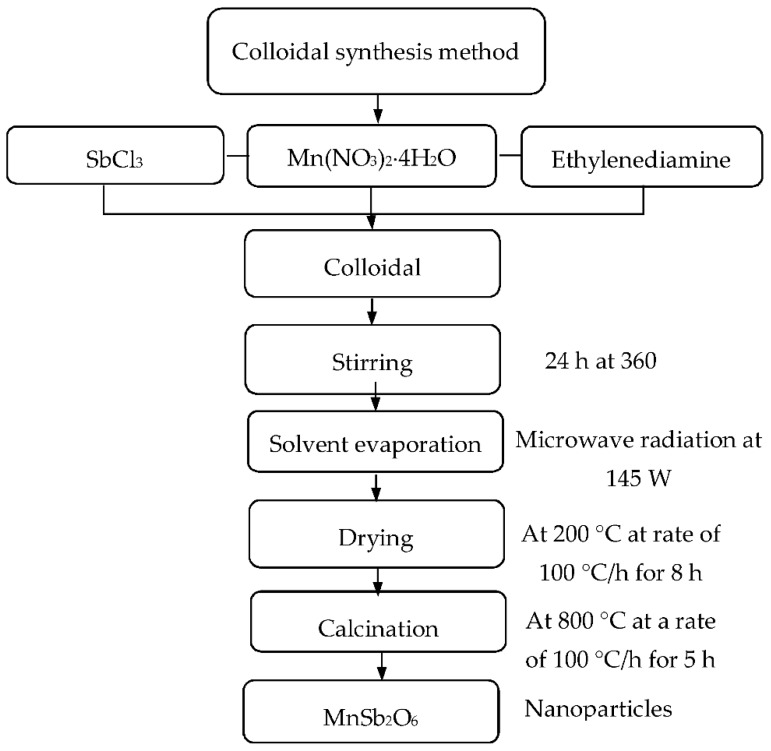
Synthesis process of the manganese antimonate nanoparticles.

The manganese antimonate nanoparticles were prepared using the microwave-assisted colloidal method. For this, 1.25 g (5 mmol) of Mn(NO_3_)_2_∙4H_2_O (Sigma-Aldrich, St. Louis, MO, USA), 2.28 g (10 mmol) of SbCl_3_ (Sigma-Aldrich), 0.5 mL (8 mmol) of ethylenediamine (Sigma-Aldrich) and ethyl alcohol as a solvent (Golden Bell, Taiwan) were used. The amounts of the Mn and Sb precursors were calculated according to the nominal composition of the MnSb_2_O_6_ (ratio Sb:Mn = 2:1). To each reagent, 5 mL of alcohol was added and then stirred for 30 min in order to obtain homogeneous solutions. Subsequently, the solutions with ethylenediamine and antimony chloride were mixed together under stirring. After that, manganese nitrate was added, obtaining a white solution, which was stirred vigorously at 360 rpm for 24 h in air at room temperature. The solvent was evaporated by making 16 applications of microwave radiation at a low power (145 W, with a duration of 1.2 min per application) using a domestic microwave oven (LG model MS1147, Guadalajara, Jal., Mexico). Subsequently, the precursor material (a white paste) was dried at 200 °C in air for 8 h and then calcined at 800 °C for 5 h at a rate of 100 °C/h by way of a muffle with programmable temperature control (Novatech, Tlaquepaque, Jal., Mexico). Figure 1 illustrates the process for obtaining the MnSb_2_O_6_ nanoparticles.

### 2.2. Physical Characterization of MnSb_2_O_6_

The crystalline phase of the antimonate was analyzed using X-ray diffraction at room temperature using a Panalytical Empyren device with CuKα radiation (λ = 1.546 Å). The diffraction was done using a 2*θ* continuous scan from 10° to 90° with 0.026°-steps at a rate of 30 s per step. The microstructure was examined using a field-emission scanning electron microscope (FE-SEM, Tescan MIRA 3 LMU system, 10 kV; Mexico City, Mexico). Size and morphology of the nanoparticles were analyzed through a transmission electron microscope (TEM, Joel JEM-ARM200F, Mexico City, Mexico) in image mode. In order to observe the individual features of the nanoparticles, powders of MnSb_2_O_6_ were placed inside a container to which methanol was previously added for the purpose of dispersing the powders. The dispersion was carried out for 10 min in an ultrasonic generator and then the powders were deposited on a 300-mesh copper grid containing a formvar/carbon membrane (Tedpella, Redding, CA, USA).

The optical properties of the MnSb_2_O_6_ were determined by means of photoacoustic spectroscopy (PAS). The equipment consisted of an Oriel Xenon lamp as the excitation source and an Oriel monochromator for recording the optical absorption spectra. Mechanically modulated radiation of 17 Hz was focused onto the optical fiber, which was connected to the PA cell. The PA signal was detected by a sensitive microphone through a 1 mm (Ø)-opening made on the cell’s wall and then amplified using the lock-in amplifier (EG&G, 5210). The PA spectra were normalized with the PA spectrum obtained from a carbon black reference. The characterization of the surface area of the MnSb_2_O_6_ powders was done by nitrogen adsorption measurements using a Belsorp-mini II analyzer (BEL Japan, Osaka, Japan). The sample was degassed and maintained under vacuum conditions for 24 h at room temperature.

### 2.3. Gas Sensitivity Tests

To carry out the gas detection tests, pellets were made from the antimonate powders calcined at 800 °C. The pellets, with a diameter of 12 mm and a thickness of 0.5 mm, were prepared by compacting 0.300 g of powders applying a pressure of 10 tons during 60 s by means of a manual-pressing-equipment (Simplex Ital Equip–25 tons). To measure changes in the material’s electrical resistance in carbon monoxide (CO) and propane (C_3_H_8_) atmospheres, two ohmic contacts were traced on the pellets using colloidal silver paint (Alfa Aesar, 99%). The pellets were placed inside a measuring chamber with a vacuum capacity of 10^−3^ torr. The partial pressure of the gases inside the chamber was controlled using a Leybold TM20 detector and the measurement of the electrical resistance was done using a Keithley 2001 multimeter.

The concentrations of carbon monoxide and propane were of 1, 5, 50, 100, 200 and 300 ppm, plus two more for propane: 400 and 500 ppm. The operating temperatures were 100, 200 and 300 °C for both gases. The sensitivity (*S*) of the pellets was estimated according to the equation [5,7,9,12,21,22]:

(1) $$S=\frac{G_{G}-G_{O}}{G_{O}},$$

where *G_G_* and *G_O_* are the conductance of the pellets (1/electric resistance) in the test gases (CO or C_3_H_8_) and air, respectively. A diagram of the device used for the gas detection tests can be found in reference [6].

## 3. Results

### 3.1. X-ray Diffraction Analysis

**Figure 2:**
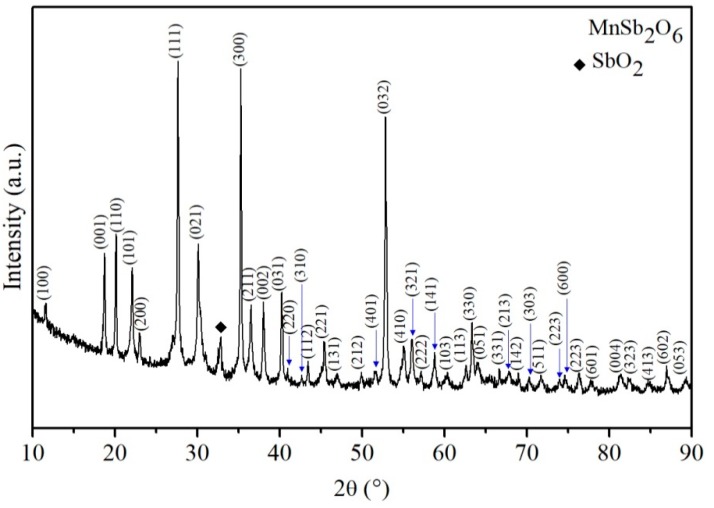
X-ray diffraction pattern of the MnSb_2_O_6_ powders calcined at 800 °C.

As expected, the reaction between the manganese nitrate and the antimony chloride, in combination with the ethylenediamine, produced the characteristic peaks of the antimonate’s crystalline phase when the precursor material was calcined at 800 °C. Temperature played a key role in promoting the reaction, giving rise to the results shown in Figure 2.

It can be clearly seen in Figure 2 that the peaks are narrow and high, which means that the material was crystalline. In addition, the width of the peaks suggests that it was composed of nanometric crystallite sizes [5,7,9]. The peaks were identified by means of the PDF No. 84-1237, showing that the oxide had a hexagonal structure [23] with cell parameters a = b = 8.8054 Å and c = 4.7229 Å and the spatial group P321 [20]. Therefore, the MnSb_2_O_6_ belongs to the antimonates family [18,24]. A secondary phase of inorganic material corresponding to SbO_2_ was also found (identified through PDF No. 65-2446), located at the point 2*θ* = 32.7°. The crystallite size (*t*) of the manganese antimonate was calculated by applying the Scherrer equation [7]:

(2) $$t=\frac{0.9\lambda }{\beta cos\theta }$$

where *λ* is the wavelength of the radiation (*λ* = 1518 nm), *θ* is the Bragg angle and *β* is the full width at half maximum (FWHM) of the diffraction peak. The calculation was carried out considering all the peaks of the main phase of the MnSb_2_O_6_ shown in Figure 2, finding that the average crystallite domain was of ~36.6 nm. The results presented in Figure 2 are consistent with other studies on the same material [25] or similar compounds belonging to the antimonates family (MSb_2_O_6_, where M = Mn, Cu, Co, Ni, among others) [1,2,3,4,20,25].

### 3.2. Scanning Electron Microscopy Analysis

**Figure 3:**
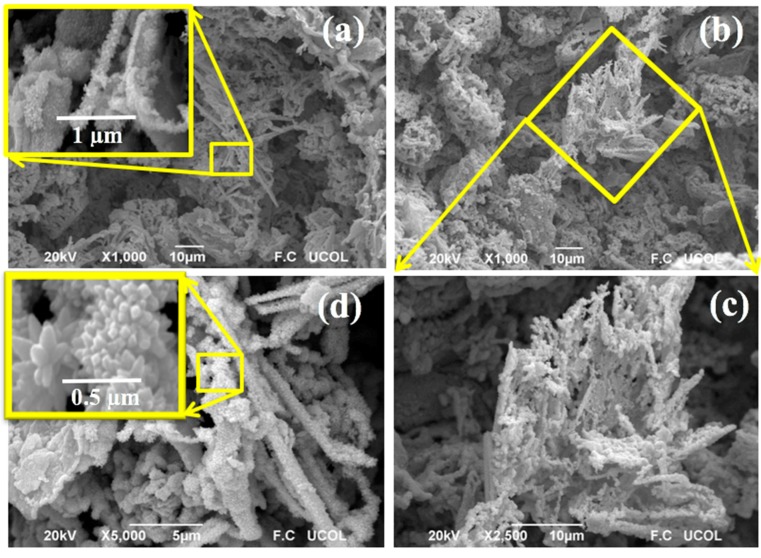
SEM photomicrographs showing the surface’s microstructure of the manganese antimonate calcined at 800 °C at magnifications: (**a**) 1000×, (**b**) 1000×, (**c**) 2500× and (**d**) 5000×.

Figure 3 shows scanning electron microscopy (SEM) images of the manganese antimonate powders calcined at 800 °C. For the analysis of the material’s microstructure, the magnifications 1000×, 2500× and 5000× were used.

**Figure 4:**
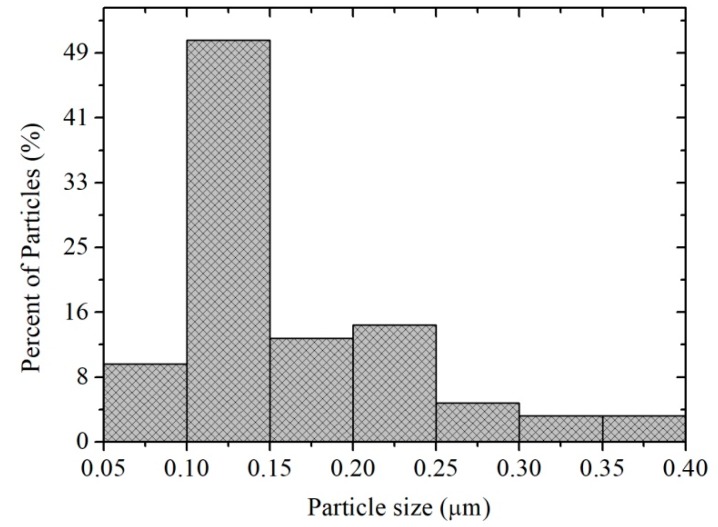
Size distribution of the particles that shape the MnSb_2_O_6_ micro-rods obtained.

In Figure 3a, the microstructural composition of the material is broadly observed. It can be clearly seen the appearance of several types of morphologies formed by irregular particles of different sizes. The type of microstructure with greater abundance in this area was due to the growth of very fine particles with an average size of ~1.3 μm. It was also possible to identify a morphology similar to micro-rods (see inserted image) with sizes in the range of 4–20 μm, with an average size of ~10.27 μm and a standard deviation of ~4.1 μm. The morphology shown in Figure 3a–c appears to be a fibrous and porous surface. Figure 3d, obtained at a higher magnification (5000×), shows that the micro-rods are formed by the agglomeration of particles (see inserted image). The size of the particles that shape the micro-rods was estimated in the range of 0.05–0.4 µm, with an average of ~0.159 µm and a standard deviation of ~0.068 µm (see Figure 4). This type of microstructure is attributed to the agglomeration of particles that grew due to the temperature and the chelating agent (ethylenediamine) employed during the synthesis process [5,7]. The porosity on the material’s surface was due to the release of gases produced during the thermal treatment of the material [26], which caused the decomposition of organic species but mainly water vapor, NO_x_ and CO_2_ [27].

The use of ethylenediamine to produce different types of nanostructures (such as nanowires and nanorods) has already been discussed in previous works [26,28]. In particular, ethylenediamine acts as a modulating agent of the microstructure, incorporating itself as a mesh in the inorganic particles, to subsequently escape from them due to the applied thermal treatment, causing the nucleation and formation of the morphology shown in Figure 3a–d.

### 3.3. Transmission Electron Microscopy Analysis and Nitrogen Adsorption Tests

**Figure 5:**
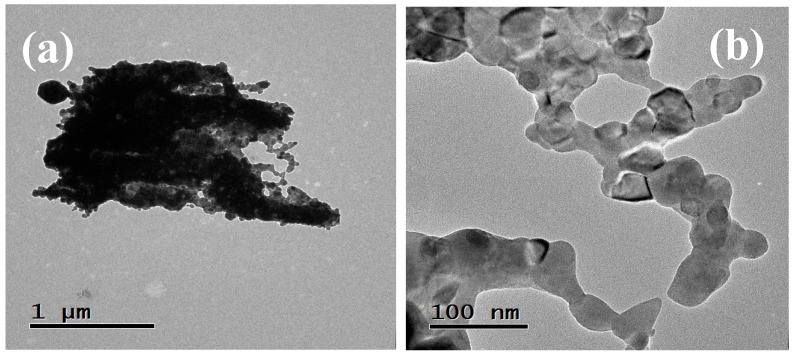

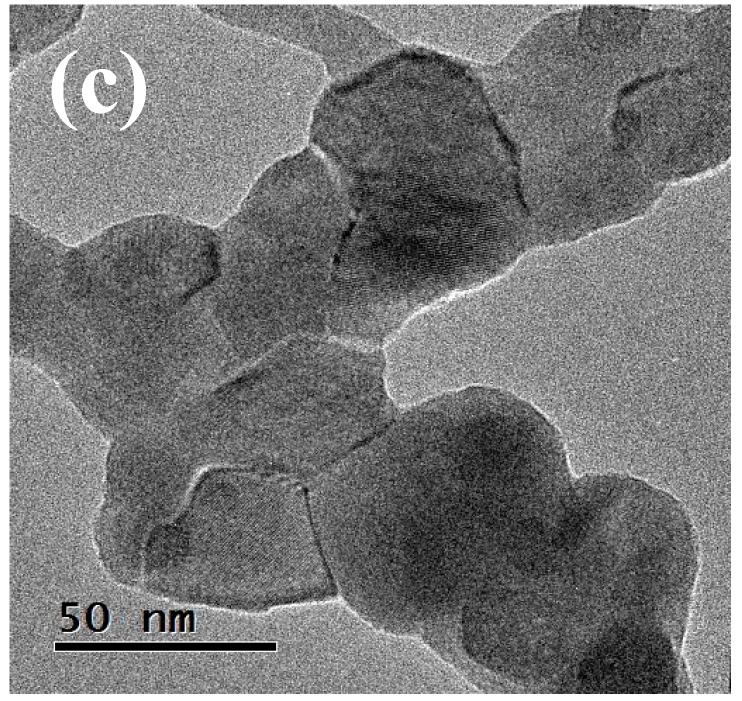
(**a**–**c**) TEM photomicrographs of the manganese antimonate nanoparticles calcined at 800 °C.

**Figure 6:**
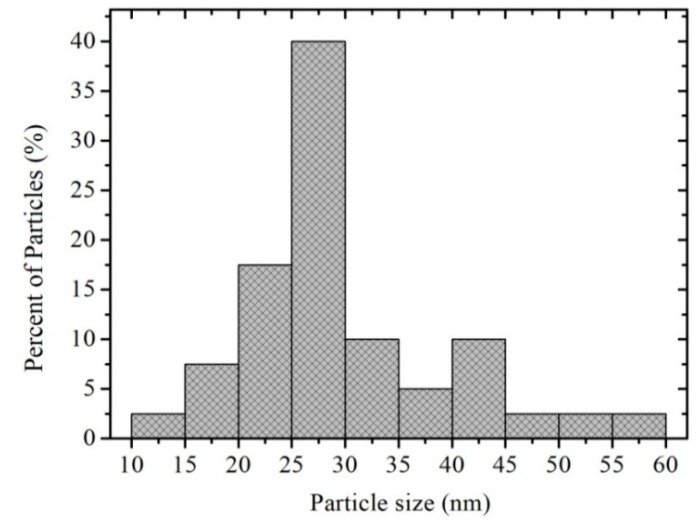
Histogram showing the nanoparticle size distribution of calcined MnSb_2_O_6_ at 800 °C.

In Figure 5, three TEM photomicrographs of the antimonate particles calcined at 800 °C are shown. These images confirm the production of nanoparticles of different morphologies and sizes. According to the analyses made in this area of the material, a large agglomeration of nanoparticles can be observed due to the effect of the calcination temperature and the residence time of the precursor material in the muffle. The dark areas depicted in Figure 5a are due to the low electron transmission that occurs because of the nanoparticles agglomeration on the material’s surface. It can be observed in Figure 5b,c that the nanoparticles are joined to each other by the formation of necks due to coalescence. The size of the nanoparticles was estimated in the range of 10–60 nm, with an average of ~29.5 nm and a standard deviation of ~9 nm (see Figure 6).

**Figure 7:**
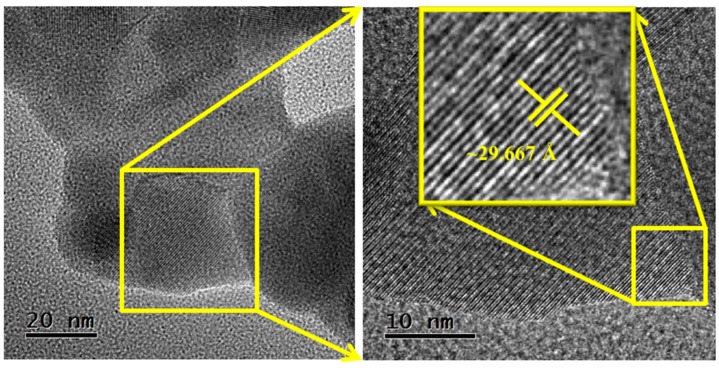
High resolution TEM (HRTEM) images showing the crystal planes of the manganese antimonate nanoparticles.

In Figure 7, high-resolution TEM (HRTEM) images of the surface of a nanoparticle (size ~34.2 nm) are shown. This demonstrates the crystalline nature of the manganese antimonate, since the crystalline planes formed on the surface of the particles joined by the necks can be observed (see the inserted image). The distance *d* between the crystalline planes was estimated on a selected area of a nanoparticle. According to this, the distance between the planes is of ~29.667 Å, which corresponds to the plane (021) of the MnSb_2_O_6_’s crystal structure, with a diffraction angle of 2*θ* = 30.097°. This result can be corroborated by the X-ray diffraction analysis shown in Figure 2.

Based on the results obtained, the microwave-assisted colloidal method is a successful way to synthesize MnSb_2_O_6_ microbars constituted by nanoparticles. It is important to mention that microwave radiation is a form of heating that offers interesting advantages in the synthesis of materials. Among them, heat can be transferred throughout the volume of the material (volumetric heating). Therefore, the heating time (some minutes in this work) is significantly reduced compared to conventional heating (usually hours). In addition, secondary reactions are reduced, increasing the reaction yield. Another important aspect is that it favors the synthesis of materials with nano-sized particles and improved physical-chemical properties [29]. These features are interesting for applications in the field of gas sensors, as we have reported here.

**Figure 8:**
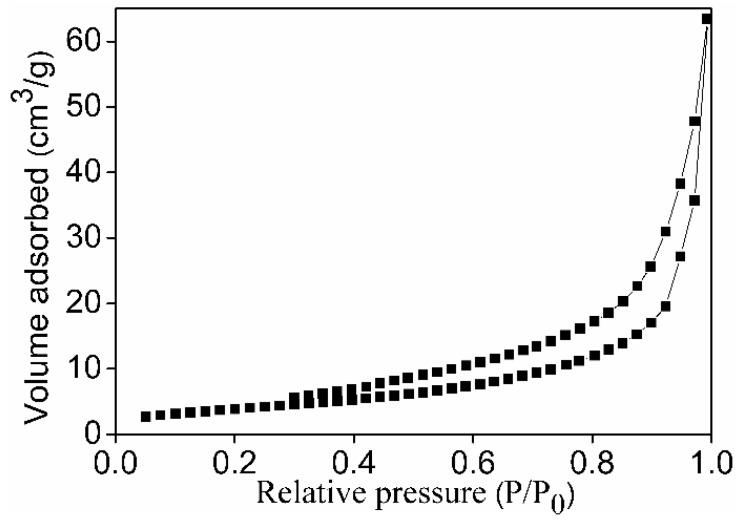
Nitrogen adsorption-desorption isotherm of the MnSb_2_O_6_ powders.

Figure 8 shows a nitrogen adsorption-desorption isotherm of the MnSb_2_O_6_ powders. The shape of the isotherm is of type II according to the IUPAC classification. This isotherm, which shows a hysteresis, is characteristic of non-porous or macroporous adsorbents. The surface area of the MnSb_2_O_6_ powders, according to the BET (Brunauer–Emmett–Teller) method, was estimated at 14.6 m^2^/g. In general, mixed oxides usually have relatively low surface areas (<10 m^2^/g) when prepared by traditional methods [27]. The synthesis of sensor materials with larger surface areas can therefore favor the adsorption of gases and, consequently, increase their response. 

### 3.4. Optical Properties Analysis

**Figure 9:**
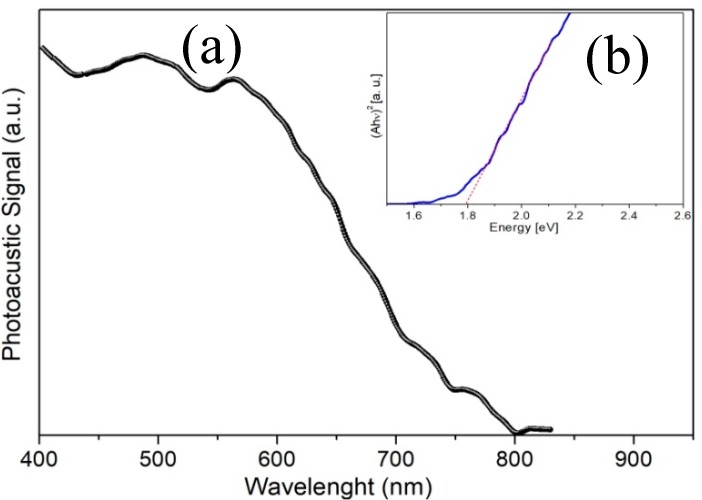
(**a**) PA spectrum as a function of the incident energy’s wavelength, (**b**) optical energy band gap for the MnSb_2_O_6_ calcined at 800 °C.

In order to determine the optical properties of the MnSb_2_O_6_, a photoacoustic spectroscopy (PAS) study has been performed. A PA spectrum as a function of the wavelength from 400 to 830 nm is shown in Figure 9a. The optical energy band gap ($E_{g}$) was determined from the high absorption region ($h\vartheta >E_{g}$, where hϑ is the incident energy). In this region, the direct allowed transition is given by the equation [30]:

(2) $$\alpha h\vartheta =A{\left(h\vartheta -E_{g}\right)}^{1/2},$$

where α is the absorption coefficient and A is a constant. The inset of Figure 9b shows the graph of ${\left(Ah\vartheta \right)}^{2}$ as a function of hϑ. The value of $E_{g}$ was estimated by the extrapolation of the dotted line with the abscissa [31,32], as shown in the inset. The value of the corresponding $E_{g}$ for MnSb_2_O_6_ was of 1.79 eV.

### 3.5. Gas Sensing Tests

#### 3.5.1. CO Analysis

**Figure 10:**
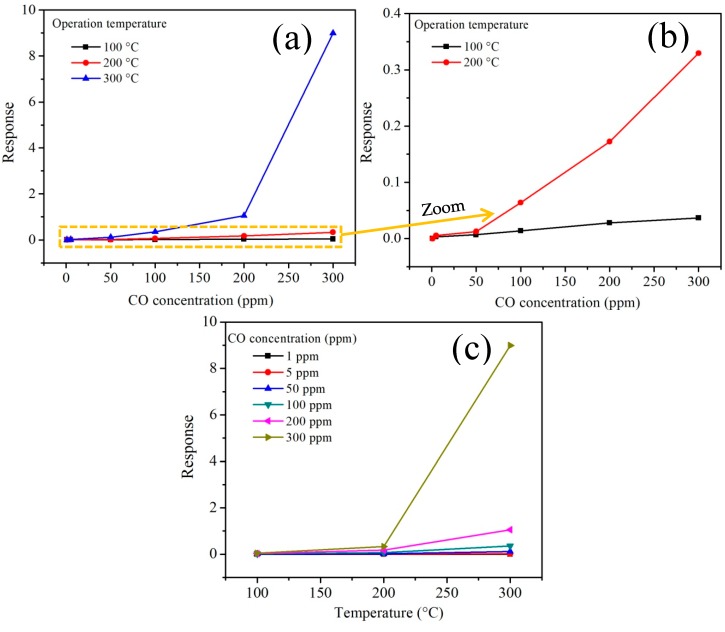
Response of the manganese antimonate nanoparticles in terms of (**a**) CO concentration, (**b**) concentration at 100 and 200 °C and (**c**) temperature.

Detection experiments in carbon monoxide (CO) atmospheres were carried out using concentrations of 1, 5, 50, 100, 200 and 300 ppm at operating temperatures 100, 200 and 300 °C. The results are shown in Figure 10. As can be seen in Figure 10a, at 100 °C the antimonate pellets showed no response (i.e., no changes in the material’s electrical resistance were recorded) at any CO concentration. However, by increasing the operating temperature to 200 °C, small increases in sensitivity were obtained by also increasing the CO concentration. The response variations were 0.0, 0.0053, 0.0125, 0.0640, 0.172 and 0.329 at concentrations 1, 5, 50, 100, 200 and 300 ppm of CO, respectively. Figure 10b shows a zoom to Figure 10a where the response of the material at 200 °C can be checked in detail. The poor response recorded at 100 and 200 °C is attributed to the fact that the thermal energy is not enough to provoke the reaction of the CO molecules with the surface of the pellets, which leads to the absence of oxygen desorption at these temperatures. In contrast, when the temperature was raised to 300 °C, a very significant increase in response was recorded (~8.98, see Figure 10c). It can be clearly seen that the sensitivity magnitude of the material depends on the increase of the concentration and the operating temperature. It has been reported that the effect of increasing the temperature and concentration of a gas like CO considerably raise the response of a semiconductor material [33,34], as in our case.

The high response recorded in CO atmospheres is caused by the increase in temperature during the detection test, contributing to an increase in oxygen desorption [35]. The mechanism that explains the interaction between the CO and the surface of the antimonate pellets is based on the ionization states of the chemisorbed oxygen on the material’s surface due to the temperature [36]. Chang [37] reports that due to the effect of temperature during the sensing tests, different oxygen species can appear. Therefore, at temperatures below 150 °C the oxygen species that dominate the most are $O_{2}^{-}$, whereas when the temperature rises above 150 °C (in our case at 300 °C) the oxygen species that emerge are $O^{-}$ and $O^{2-}$ [36,38], which are more reactive than those appearing below 150 °C [36].

**Figure 11:**
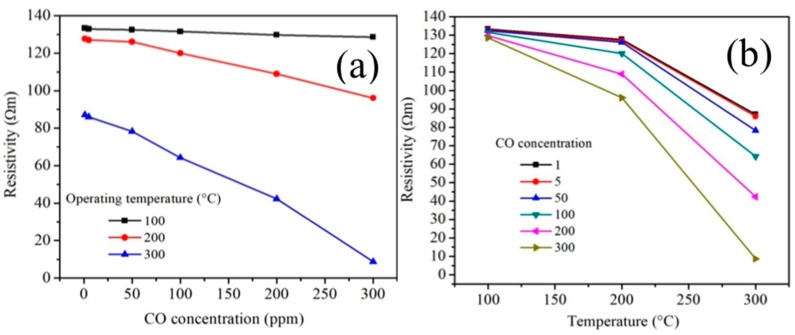
Electrical resistivity of the MnSb_2_O_6_ pellets as a function of (**a**) CO concentration and (**b**) operating temperature.

The results of the electrical resistivity (or response) of the antimonate nanoparticles in the presence of CO at the given operating temperature are shown in Figure 11a,b. The calculation was done using the equation ρ=R A/t [39] where *R* is the electrical resistance in the test gas and *A* and *t* are the cross-sectional area and the thickness (0.5 mm; Ø = 12 mm) of the pellets, respectively.

As can be observed in Figure 11, with the increase of CO concentration, the resistivity of the MnSb_2_O_6_ nanoparticles decreased. In addition, the effect of temperature played a very important role in the response of the material. As the operating temperature increased (from 100 to 300 °C), the MnSb_2_O_6_ pellets exhibited a very significant decrease in electrical resistivity. This behavior is typical of a semiconductor material used as a gas sensor [34,39]. Thus, at 100 °C, the highest resistivity of the material was recorded. When the temperature increased to 200 °C, the pellets showed a slight decrease in electrical resistivity. In addition, with the increase in temperature, the material showed inflection points on the resistivity at 200 °C. The poor response obtained at these temperatures (100 and 200 °C) is largely due to the fact that the thermal energy was not enough to provoke the reaction of the CO molecules with the oxygen present on the pellets’ surface [34]. On the contrary, when the temperature was raised to 300 °C, the values of the electrical resistivity in CO atmospheres decreased very significantly. The values found at this temperature were 87.0, 85.88, 78.19, 64.18, 42.26 and 8.71 Ωm. The results shown in Figure 11a,b are consistent with similar oxides tested in the same gas [34,39,40].

#### 3.5.2. C_3_H_8_ Analysis

**Figure 12:**
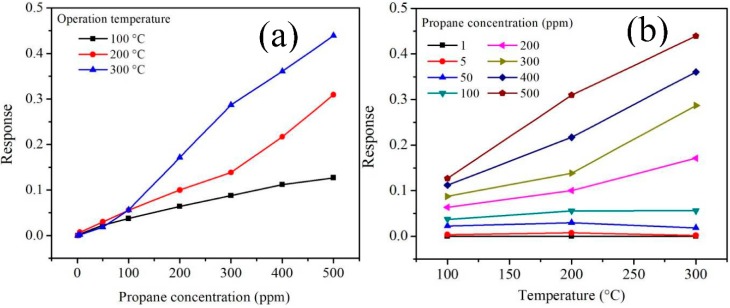
Response of the manganese antimonate nanoparticles as a function of (**a**) propane concentration and (**b**) operating temperature.

The tests in propane atmospheres (C_3_H_8_) were carried out at different concentrations (1–500 ppm) and operating temperatures (100, 200 and 300 °C). The results under these working conditions are shown in Figure 12.

As expected, as the temperature and concentration of the test gas increased, the values of the electrical resistance changed, causing increases in the material’s sensitivity magnitude. The increase in the response of the MnSb_2_O_6_ nanoparticles is mainly associated with the increase in the number of propane molecules that react with oxygen chemisorbed on the material’s surface due to the temperature [9,26,27]. Different authors have reported that variations in electrical resistance occur due to the increase in temperature, causing the sensitivity magnitude to increase [5,6,8,26,35,37]. We observed that at 100 and 200 °C, the thermal energy was not enough to generate the oxygen desorption reaction on the pellets’ surface, which led to low changes in the material’s electrical resistance, that is, a poor response was obtained. On the other hand, at a higher temperature (300 °C), more oxygen species were produced, causing a high interaction between the propane molecules and the surface of the pellets, with an increase in the sensitivity magnitude. In particular, the increase in the response at 100 and 200 °C was respectively of ~0.127 and ~0.309 at 500 ppm of propane. The maximum response was of ~0.439, corresponding to 500 ppm of C_3_H_8_ at 300 °C.

The mechanism that accounts for the interaction between the propane molecules with the surface of the MnSb_2_O_6_ pellets has not been fully explained [27]. However, it has been reported that some of the factors that affect the chemisorption of oxygen are the particle size and the morphology since if the size is less than twice the thickness of the charged outer layer $\left(L_{S}\right)$, the oxygen species that are adsorbed are $O^{-}$, which leads to an increased response [40,41]. Other authors have suggested that the propane molecules react with the chemisorbed species $O^{-}$ by producing CO_2_, water vapor and thereby a release of electrons on the material’s surface [27,42]. This phenomenon causes changes in the electrical resistance of the solid material and with it a very significant increase in the response (see Figure 12a,b).

**Figure 13:**
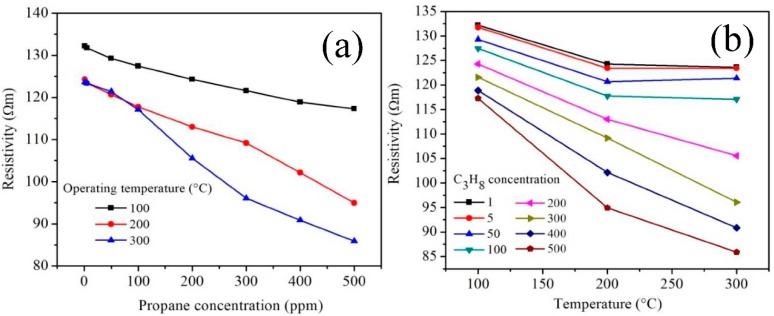
Electrical resistivity of the MnSb_2_O_6_ pellets as a function of (**a**) propane concentration and (**b**) operating temperature.

As in the case of CO, the electrical resistivity (*ρ*) in propane atmospheres at temperatures of 100, 200 and 300 °C was estimated using the equation *ρ = RA/t* [39]. The results are presented in Figure 13 as a function of propane concentration and operating temperature. As shown in Figure 13a,b, with the increase in temperature, the material’s resistivity decreased in a similar manner to the case of CO. We have confirmed that by injecting the different concentrations of propane (1–500 ppm) into the measuring chamber, the pellets’ resistivity decreases significantly. This is attributed to the fact that with the increase of the operating temperature, the mobility of the solid material’s charge carriers is more active on the pellets’ surface [34,43], which contributes to the increase of the material’s electrical conductivity proportionally to the reduction of its electrical resistivity in C_3_H_8_ atmospheres [44]. In addition, the increase in the concentration of propane favored the low electrical resistivity obtained at high temperatures (in our case at 200 and 300 °C). This means that with the increase of the operating temperature, the kinetic activity of the propane gas molecules increased, leading to the results shown in Figure 13a,b. It has been reported that for the response of a material such as the one used in this work, the changes in electrical resistivity depend on the sensor type employed [34] since the interaction between the gas molecules and the chemisorbed oxygen varies depending on the geometry of the sensor (e.g., pellets or films) [34,39,44]. In our case, the variation of the pellets’ electrical resistivity at 300 °C was of 123.52, 123.39, 121.36, 17.08, 105.54, 96.05, 90.85 and 85.88 Ωm. This trend generally occurs in semiconductor oxides when exposed to gases at high temperatures [34,43,45,46,47,48], as in our case (see Figure 13a,b).

By comparing our results with similar metal oxides that have been tested as potential gas sensors, our manganese antimonate pellets showed a better response. For example, in reference [49], the metal oxide SnO_2_ was prepared to study its ability to detect propane atmospheres, finding a response of ~0.1 at 300 °C using films with a thickness of 50 nm. Same reference reports a maximum response of ~0.35 in the same operating conditions using films with a thickness of 100 nm. In reference [50] ZnMn_2_O_4_ nanoparticles were synthesized for studying their detection properties in CO atmospheres. Using oxide pellets, they obtained a response of ~1.55 at a temperature of 300 °C and a concentration of 300 ppm of CO. Meanwhile, reference [27] reports a maximum response of 7.2 at 300 °C and 300 ppm of CO. In addition, our research group recently reported that pellets of the trirutile-type oxide CoSb_2_O_6_ showed a maximum response of ~7 at 350 °C and 300 ppm of CO [6] and of ~4.14 at 300 °C and 300 ppm of CO [51]. 

In summary, the manganese antimonate pellets were clearly sensitive to changes in their electrical properties in gaseous atmospheres of carbon monoxide and propane. In addition, it was confirmed that the response of the material is affected by the type of gas and its concentration, as well as by the operating temperature. At this point, it is important to stress the impact of the nanostructures on the gas response. It is a known fact that the structure of the sensitive layer can also affect the performance of the sensing materials due to the nanometer size of the particle [41,52]. In practice, nanostructured materials present an optimal combination of properties that make them an excellent choice for application as gas sensors. Among such properties, the structural stability and the electro-physical properties stand out, as well as an inexpensive design technology and a well-developed surface [53]. In addition, the surface area increases when the particle size decreases (~14.6 m^2^/g in the case of the MnSb_2_O_6_), thus increasing the number of potentially active catalytic sites, which favors a high adsorption of CO and C_3_H_8_ on the surface, contributing in this way to obtain changes in the electrical resistance and, consequently, to the improvement of the response to these gases [6]. That is, its ability to detect several gas concentrations increases greatly, allowing in turn the detection of low gas concentrations (1 ppm in this case) [9,54]. The thickness of the charged outer layer is a function of the concentration of charge carriers in the volume of the material. Therefore, three conduction mechanisms can appear depending on the crystal size (*D*) [5,45,55,56,57]: (1) If $D\gg 2L_{S}$, the conductivity will be limited to the Schottky barrier at the particle boundary; in this case, the response is practically independent of the crystal size; (2) if $D=2L_{S}$, the crystal size is comparable to $2L_{S}$ and the conductivity will depend on the formation of necks between the crystals; in this case, the dependence of the gas response on the crystal size is defined; (3) if $D<2L_{S}$, the whole crystal will be involved with the charged outer layer and the conductivity will depend only on the crystal size. Therefore, if it is possible during the synthesis process to obtain particle sizes smaller than 100 nm (in our case it was of ~30 nm), the electronic conduction will be through the surface of the sensor [45,54], which will lead to a high sensitivity, as occurred in this work.

## 4. Conclusions

Nanoparticles of manganese antimonate (MnSb_2_O_6_) were successfully synthesized using the low-power microwave-assisted colloidal method. This process is economical, easy to use and very efficient for producing particles with sizes below 100 nm. The average size of the MnSb_2_O_6_ nanoparticles was estimated at ~29.5 nm, while the surface area of the oxide powders was of 14.6 m^2^/g. The optical energy band of the crystalline phase obtained at 800 °C was estimated at 1.79 eV. Pellets made from the material’s nanoparticles showed to be highly sensitive to carbon monoxide (CO) and propane (C_3_H_8_) atmospheres at different operating temperatures. The optimal functioning of the sensor was at a temperature of 300 °C and at concentrations of 300 ppm of CO and 500 ppm of C_3_H_8_. The maximum sensitivity recorded in these atmospheres was of ~8.98 (CO) and ~0.439 (C_3_H_8_), respectively. According to our results, nanoparticles of manganese antimonate can be considered as an excellent sensor of carbon monoxide and propane atmospheres.
